# Enablers and barriers to implementing collaborative care for anxiety and depression: a systematic qualitative review

**DOI:** 10.1186/s13012-016-0519-y

**Published:** 2016-12-28

**Authors:** Gritt Overbeck, Annette Sofie Davidsen, Marius Brostrøm Kousgaard

**Affiliations:** The Research Unit for General Practice and Section of General Practice, Institute of Public Health, University of Copenhagen, København, Denmark

**Keywords:** Collaborative care, Shared care, Implementation, Enablers, Barriers, Anxiety, Depression, Qualitative review

## Abstract

**Background:**

Collaborative care is an increasingly popular approach for improving quality of care for people with mental health problems through an intensified and structured collaboration between primary care providers and health professionals with specialized psychiatric expertise. Trials have shown significant positive effects for patients suffering from depression, but since collaborative care is a complex intervention, it is important to understand the factors which affect its implementation. We present a qualitative systematic review of the enablers and barriers to implementing collaborative care for patients with anxiety and depression.

**Methods:**

We developed a comprehensive search strategy in cooperation with a research librarian and performed a search in five databases (EMBASE, PubMed, PsycINFO, ProQuest, and CINAHL). All authors independently screened titles and abstracts and reviewed full-text articles. Studies were included if they were published in English and based on the original qualitative data on the implementation of a collaborative care intervention targeted at depression or anxiety in an adult patient population in a high-income country. Our subsequent analysis employed the normalization process theory (NPT).

**Results:**

We included 17 studies in our review of which 11 were conducted in the USA, five in the UK, and one in Canada. We identified several barriers and enablers within the four major analytical dimensions of NPT. Securing buy-in among primary care providers was found to be critical but sometimes difficult. Enablers included physician champions, reimbursement for extra work, and feedback on the effectiveness of collaborative care. The social and professional skills of the care managers seemed critical for integrating collaborative care in the primary health care clinic. Day-to-day implementation was also found to be facilitated by the care managers being located in the clinic since this supports regular face-to-face interactions between physicians and care managers.

**Conclusions:**

The following areas require special attention when planning collaborative care interventions: effective educational programs, especially for care managers; issues of reimbursement in relation to primary care providers; good systems for communication and monitoring; and promoting face-to-face interaction between care managers and physicians, preferably through co-location. There is a need for well-sampled, in-depth qualitative studies on the implementation of collaborative care in settings outside the USA and the UK.

**Electronic supplementary material:**

The online version of this article (doi:10.1186/s13012-016-0519-y) contains supplementary material, which is available to authorized users.

## Background

Mental health problems like anxiety and depression are a serious burden for patients and health-care systems across the world [1, 2]. The majority of patients with anxiety and depression are treated in primary care, and in order to improve their treatment, collaborative care models have been developed inspired by the chronic care model [3, 4]. Collaborative care involves an intensified and structured collaboration between primary care providers and health professionals with specialized psychiatric expertise [2, 5]. The model’s multi-professional approach to patient care usually comprises a primary care physician; a case/care manager (e.g., psychiatric nurse) and/or a mental health specialist; a structured management plan (for instance, manuals for psychological intervention and sometimes for medication management); systematic patient follow-up; and enhanced communication between health professionals (e.g., shared medical records, team meetings, and supervision) [1]. However, variations exist in the exact composition and enactment of the central elements, e.g., concerning the degree of structure of the management and educational plans and manuals that care managers should follow, the means of treatment delivery (telephone-based or face-to-face or a combination hereof), the method of supervision, and the use of stepped care approaches to treatment [1]. Notwithstanding such variations, collaborative care is widely considered to be an evidence-based concept for improving the quality of mental health care [3]. Thus, a Cochrane Review from 2012 based on 79 collaborative care trials found evidence that collaborative care for depression and anxiety was more effective than usual care in improving treatment outcomes for depression and anxiety (as measured by an observer or by patient self-report). The effects were significant up to 24 months both in terms of measures of depression or anxiety. In terms of secondary outcomes, collaborative care increased the rates of antidepressant use and improved mental health quality of life and patient satisfaction, also up to 24 months. Most trials focused on depression and most were conducted in the USA [1], where health insurance systems can create a patient selection bias that may reduce the ability to generalize to countries with broader health coverage [6]. However, studies in other health systems have found effects of collaborative care comparable to those in the US studies [1]. Thus, the Collaborative Depression Trial (CADET) in the UK—one of the largest collaborative care trials outside the USA—showed effect in improving depression for up to 12 months [7]. However, CADET investigators had hoped for even larger effects and recommend that future studies should concentrate on optimizing the design and delivery of collaborative care models to further improve outcomes [7]. In the UK, CADET has been succeeded by efforts to integrate collaborative care into routine clinical practice and to focus on patients suffering from both depression and long-term physical conditions, cf., the COINSIDE trial [8]. Collaborative care is a complex intervention consisting of several active components and requiring the enrolment of various professional actors from different sectors. The diffusion and implementation of collaborative care can therefore be difficult, and hence, it is important to understand the specific factors which affect implementation [3, 9]. Qualitative systematic reviews are recognized as a method for gathering and synthesizing existing knowledge of implementation processes and challenges [10]. Against this background, we performed a qualitative systematic review of the enablers and barriers to implementing collaborative care for patients with anxiety or depression.

The analytical approach of the review was based on the normalization process theory (NPT) which is a theory for studying the implementation and embedding (normalization) of complex interventions in organizations [11, 12]. NPT is a theoretical extension of the normalization process model, which was developed on the basis of various qualitative studies of change processes in health care, and first explicitly applied to the case of implementing telemedicine services [13]. Since the development of NPT, the theory has been used to study implementation across a wide range of topics and settings in health care (e.g., [14–18]). NPT focuses on the implementation efforts of the involved actors, and on the factors which inhibit or facilitate normalization. According to NPT, the mechanisms of implementation processes can be captured through four theoretical dimensions [11, 12]: (1) coherence: how actors understand and make sense of an intervention which is to be implemented in the organization; (2) cognitive participation: how actors engage in the implementation process; (3) collective action: how the intervention is enacted in daily practice and how the actors’ skills and organizational resources connect to the intervention and influence implementation; and (4) reflexive monitoring: how the intervention is assessed formally and/or informally by actors as the implementation of the intervention gets underway. Such assessments of the consequences of an intervention may affect the actors’ coherence, their cognitive participation, and lead to changes in the operationalisation of the intervention, i.e., collective action. We employed NPT in the review because it presents a framework for ordering and describing the results from different implementation studies. NPT has previously been applied in systematic reviews [19, 20] and overviews of systematic reviews [21, 22].

## Methods

In reporting this review, we used the enhancing transparency in reporting the synthesis of qualitative research (ENTREQ) standards for enhancing transparency when synthesizing qualitative research [23].

### Search strategy and sources

The aim of the study was to perform a systematic review of qualitative studies on the enablers and barriers to implementing collaborative care for patients with anxiety and depression. We searched for original research published in indexed journals. A comprehensive search strategy was developed in cooperation with a research librarian. First, we made an expansive list of possible relevant keywords related to the generic terms “implementation,” “collaborative care,” “depression,” “anxiety,” and “qualitative study.” Second, we made an initial search, imported titles to an EndNote database, and checked whether relevant studies already known to us were present in the search results. Since some relevant studies had not been identified, we adjusted the search string and performed a new search to strengthen comprehensiveness. The final search was conducted between October 2 and October 8, 2015, in the following databases: EMBASE, PubMed, PsycINFO, CINAHL, and ten ProQuest databases (see Additional file 1 for the PubMed search string and specification of ProQuest databases). To further check the comprehensiveness of the electronic search (and potentially complement it), we manually searched the reference list “References to studies included in this review” in the Cochrane Review on collaborative care [1] (pp. 27–47). This list contains the RCT studies included in the Cochrane Review as well as references to published work associated with them (for example, process evaluations of the RCT).

### Study selection

All three authors independently screened the titles and abstracts of all search hits and then collectively agreed on the articles to read in full text. Subsequently, all authors independently assessed the selected articles, and then agreed on which articles to include in the review.

### Inclusion and exclusion criteria

Our inclusion criteria were as follows:

1. Studies published in English based on original qualitative data (mixed methods studies were included if the qualitative element contributed data about facilitators and barriers)
2. Studies focusing on implementation of a collaborative care intervention (pilot or full scale) targeting depression (not bipolar), major depression, dysthymia, or anxiety in an adult patient population in a high-income country (the latter criterion is applied to limit the degree of variation in the context of implementation)

In order to qualify as collaborative care, the intervention described should include a structured treatment program of collaboration between: (1) a primary care physician/general practitioner/family physician (PCP), (2) a care manager/case manager (CM), usually a nurse, social worker, or psychologist trained to coordinate and follow up on treatment and often performing brief behavioral treatment, and (3) a mental health specialist (MHS) who provides supervision to CMs and/or PCPs [5, 24]. In two cases, we contacted the relevant authors with questions regarding the descriptions of the various interventions.

With these criteria, we excluded the following: (1) studies not published in English, review studies, and studies using only quantitative data; (2) studies of interventions falling outside our definition in terms of intervention design, patient population, and setting; (3) studies only focusing on intervention outcomes even though qualitatively, e.g., patient experiences; and (4) studies only investigating health professionals’ attitudes or expectations prior to implementation. Although such studies may provide relevant information when planning an intervention, they do not provide knowledge on the actors’ perceptions and experiences of the actual implementation.

### Quality assessment

Two researchers (GO and AD) employed the consolidated criteria for reporting qualitative research checklist (COREQ) to perform an assessment of the quality of transparency in the included studies [25]. Questions that arose from this process were discussed and resolved by all authors. We neither excluded nor did we give special priority to any studies during the analysis based on the COREQ quality assessment.

The COREQ checklist was originally developed to support the quality of reporting of individual qualitative studies [25]. However, in the ENTREQ statement, it is suggested that COREQ could be used as a quality assessment tool in the synthesis of qualitative research [23], and COREQ has subsequently been used as such in a number of qualitative reviews [26–28]**.**

### Data extraction

For all included papers, we extracted data on study characteristics, collaborative care model characteristics, and findings about barriers and enablers to implementation.

### Synthesis of results

Initially, three papers were selected for individual pilot coding by all three reviewers in order to identify the barriers and enablers described, which could either be labeled explicitly as such in the papers or be categorized as such based on our interpretation. Having discussed the coding results and reached a consensus, we coded the findings from all papers. Subsequently, two authors (GO and MBK) individually categorized barriers and enablers according to the dimensions of the framework offered by NPT, and then discussed and agreed on this categorization.

## Results

### Study selection

**Fig. 1:**
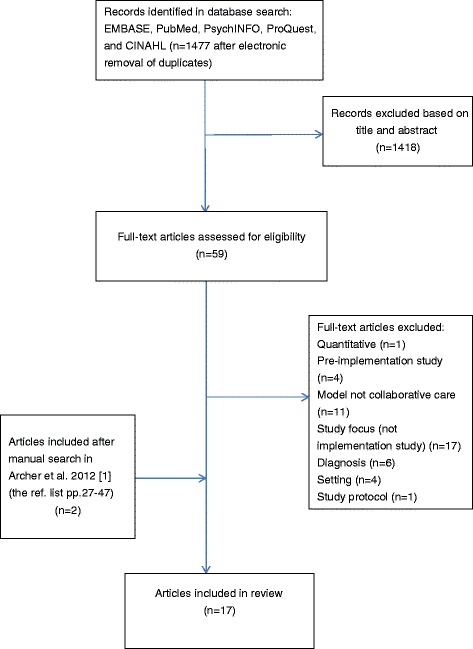
Flow chart of study selection

As shown in the flowchart (Fig. 1), 17 studies were selected for inclusion in this review. The manual search in the Cochrane Review [1] (pp. 27–47) yielded two relevant studies [29, 30] which were not recovered by the electronic search.

### Study characteristics

The characteristics of the included studies are shown in Table 1.

**Table 1:** Study characteristics

Author, name of intervention study, country	Disease	Aim of study	Data collection methods	Respondents	Data analysis/theoretical framework
Gask et al. [42], CADET, UK	Depression	To explore the work that “needs to be done to make a collaborative care intervention for depression in primary care both workable and integrated into routine practice”	Focus groups, one-to-one interviews	49: 12 PCPs 4 Clinical psychologists 4 Practice nurses 4 Psychiatrists 14 Mental health workers 11 patients	Normalization process model (NPM)
Coupe et al. [9], CADET, UK	Depression	“To explore to what extent CC impacts on professional working relationships, and if CC for depression could be implemented as routine in the primary care setting.” “To identify barriers and facilitators to the successful implementation of CC”	Face-to-face interviews with CM and managers Telephone interviews with GPs	26: 6 CMs, 5 Supervisors from research team, 15 GPs	Thematic analysis and theory-driven analysis using normalization process theory (NPT)
Knowles et al. [37], COINSIDE, UK	Depression and long-term conditions	To explore (a) the extent to which “collaborative care principles and modes of working were implemented in routine care…” and to (b) “Employ NPT as a conceptual model to identify barriers and facilitators to the adoption and integration of collaborative care…”	Face-to-face semi-structured interviews	23: 6 Case managers 17 Practice nurses	Thematic analysis and normalization process theory (NPT)
Knowles et al. [6], COINSIDE, UK	Depression and long-term conditions	“…to examine: a) How the collaborative care model was implemented by usual care providers in a UK setting. b) How patients and providers understood and experienced the integration of mental and physical health care.”	Semi-structured interviews	61: 11 PWPs 12 PNs, 7 GPs 31 Patients	The constant comparative method
Byng et al. [33], The Mental Health Link intervention (MHL), UK	Patients with long-term mental illness	To investigate how the MHL intervention “had its effects and how the process evaluation adds meaning to the results of the trial.”	Individual and group interviews	49: 21 GPs, 8 Community mental health workers, 7 Practice managers, 4 Mental health managers, 3 Practice nurses, 2 Psychiatrists, 1 Practice counselor 1 Facilitator	Case study using the realistic evaluation framework
Curran et al. [32], CALM, USA	Anxiety	To identify the facilitators and barriers to implement and sustain CALM	Qualitative interviews	61: 14 Anxiety clinic specialists (ACS) 13 Primary care nurses 18 Primary care administrators 16 Primary care clinic administrators	Content analysis. Coding in three levels: 1: macro themes identified, 2: subcoding identifying barriers and facilitators, 3: interpretation
Eghaneyan et al. [34], (Collaborative care in a community health center), USA	Depression, anxiety (in a low-income, uninsured Latino population)	“To examine the implementation of a collaborative care model…” and “to identify perceived barriers…”	Semi-structured interviews	7: 1 Care manager 3 PCPs 1 Nursing director 1 Project manager 1 CEO	Grounded theory approach. Two-leveled coding
Whitebird et al. [40], DIAMOND, USA	Depression	To identify the care model factors that were key for successful implementation of collaborative depression care	Mixed methods study: Group interviews plus “quantitative measures of patient activation and 6-month remission rates”	42 Clinics. The exact number of respondents is not stated. Present at the interviews were as follows: “…the project lead, care manager and PCP champion. Other staff encouraged to attend were other physicians, the consulting psychiatrist, and the quality improvement lead”	“Following each site visit, ICSI staff completed a structured qualitative narrative to document their assessment of factors affecting implementation […] Summaries were then prepared by the ICSI site-visit teams and were reviewed by the entire study team”
Sanchez et al. [35], IBH (Integrated Behavioral Health), USA	Depression and anxiety (in a low-income, uninsured adult population)	How a collaborative care model for the treatment of depression works	In-depth individual interviews	4: 1 Care manager 1 PCP champion 1 Psychiatrist 1 Director	Analysis was partly guided by pre-developed propositions but “allowed for analytical flexibility and identification of new themes”
Oishi et al. [29], IMPACT, USA	Late life depression	To explore how “’integration’ was achieved”, and to suggest “factors to consider when disseminating the model into real life settings”	Focus groups (2), semi-structured telephone interviews	11 DCSs (care managers)	Thematic analysis
Blasinsky et al. [41] IMPACT, USA	Major depressive disorder or dysthymia (older adults)	To investigate the sustainability of collaborative care in primary care	Semi-structured telephone interviews, documents describing the intervention, and site visits	Telephone interviews with 15 informants from 7 clinics: the principal investigator, co-principal investigator, depression care specialist (care manager), supervising psychiatrist, primary care physicians, program coordinator, and recruiter or screener	Not stated
Palinkas et al. [39], MDDP (multifaceted depression and diabetes program for Hispanics), USA	Depression and diabetes	To examine “perceptions of barriers and facilitators associated with implementation and sustainability”	Individual semi-structured interviews and focus groups	36: 5 Physicians (of which 3 were also clinic directors or associate directors) 9 Nurses 3 Nurse practitioners 19 Patients	Grounded theory approach
Huang et al. [36], MHIP, USA	Depression (high-risk mothers)	To “explore aspects of the collaborative care program associated with successful treatment of depressed mothers served in a collaborative care program as well as barriers to such successes.”	Focus group interview	6 Care managers	Thematic analysis
Tai-Seale et al. [43], PCMH (Primary Care Mental Health Initiative), USA	Depression (veterans)	To “examine the effects of collaborative care on patient and primary care provider (PCP) experiences and communication during clinical encounters”	Audio recordings of 10 patient visits and a self-administered questionnaire	6 PCPs	Qualitative analyses of transcripts using a pre-structured guide divided into six questions
Nutting et al. [30], RESPECT-D, USA	Depression	“To understand the characteristics of organizations and the intervention components that were associated with implementation and dissemination”	Telephone interviews	91: 24 Program managers (including quality improvement staff), 7 Mental health specialists, 18 Care managers, 42 Clinicians (“Most of the participating clinicians were family physicians, with only a few general internists, nurse-practitioners, and physician’s assistants”)	Data analysis in three waves focused on emerging themes
Nutting et al. [38], RESPECT-D, USA	Depression	To examine the barriers to adopting depression care management among primary care clinicians	Semi-structured telephone interviews	91: 24 Program managers 18 Care managers 7 Mental health specialists 42 Clinicians (“Most of the participating clinicians were family physicians, with only a few general internists, nurse-practitioners, and physician’s assistants”)	Data analysis in four waves focused on emerging themes
Wozniak et al. [31], TeamCare Intervention, Canada	Diabetes and depression	To evaluate the implementation collaborative care model in community-based primary care networks (PCNs)	In-person or telephone interviews, reflections of the research team during the intervention and systematic documentation (e.g., standardized checklist, field notes, and meeting minutes). The PCN managers completed a standardized checklist at baseline The researchers documented their observations of and reflections on implementing TeamCare in each PCN, using a focus group format	14 PCN staff (23 interviews) and 7 specialists (13 interviews)	Content analysis using the RE-AIM framework as well as a more inductive approach

With one exception [31], all of the collaborative care interventions in the included studies were carried out in the USA (*n* = 11) or the UK (*n* = 5) (with some interventions associated with more than one qualitative study). Also with one exception [32], all of the studied interventions targeted some kind of depressive disorder (either exclusively or in combination with other mental or physical diagnoses). Only four interventions dealt with anxiety, of which three also included depression [32–35].

There was much variation across studies concerning the level of information on the context of implementation, the specific elements of the collaborative care intervention, and of any specific implementation strategies employed. Many studies did not present detailed descriptions of these issues.

There was some variation in the aims of the included studies. Nine studies aimed to identify barriers and/or facilitators to the implementation of collaborative care [9, 30, 32, 34, 36–40]. Some studies stated their aim more broadly in terms of exploring the implementation [6, 31] or the sustainability of collaborative care [41], how change occurred [33], or the work needed to integrate collaborative care in practice [42].

Interviews were the dominant data collection method and were used in all but one study [43]. They were performed as individual face-to-face interviews, and/or telephone interviews, and/or group interviews or focus groups. No studies used participant observations, although one study used audio recordings from patient visits [43]. One study used a mixed methods design combining qualitative and quantitative data [40]. The average number of interview respondents was around 30—ranging from four respondents in the smallest study to 91 in the largest. Two studies did not state the exact number of respondents [31, 40]. The most frequent types of respondent in the studies were CMs, PCPs, and then practice staff (mostly nurses), MHSs, and managers/administrators. Less than half of the studies included respondents from all of the three central actor categories referred to in our definition of collaborative care (PCP, CM, MHS), and five studies did not include PCPs as respondents.

The analytical and theoretical approaches of the studies varied. Most studies were inductive or semi-inductive, and several used some form of thematic analysis. Four studies applied an implementation theoretical framework: Wozniak et al. [31] used RE-AIM [44, 45], Coupe et al. [9] and Knowles et al. [37] used NPT, and Gask et al. [42] used the normalization process model, the precursor to NPT [13].

### Quality assessment

We assessed all studies using the COREQ checklist [25] to gauge the explicitness and comprehensiveness of reporting. The checklist has 32 items divided into three domains: (1) research team and reflexivity, (2) study design, and (3) analysis and findings.

The results of our assessment are presented in Additional file 2. Among the 17 studies included in the review, the number of items reported on ranged from 7 to 21 (of 32), with a mean of 15.5 and a median of 15. However, only seven studies reported on more than half of the 32 COREQ criteria. The lowest rate of reporting was in domain 1 where several items were reported on by very few studies. In domain 2, items on sampling, theory, and method were reported on by most studies while fewer studies reported on non-participation (if/why potential respondents refused to participate). Reporting quality varied on issues of data collection where only four studies discussed the question of data saturation. In domain 3, the frequency of reporting was quite varied for the items on data analysis whereas most articles scored well on the presentation of findings. However, when comparing the thickness of descriptions of enablers and barriers to implementation, we found that there was much variation between studies. While some studies provided more elaborated analysis integrating descriptions and quotations, other studies provided only cursory descriptions. A few studies relied mostly on thematic headlines supported by a few quotations without further analysis. Also, in a few studies, some of the results were reported in an indirect manner as when non-PCP respondents speculated on barriers among PCPs.

### Synthesis of results

The results are presented according to the four dimensions of NPT (see also Additional file 2).

### Coherence

Generally, the studies presented few detailed findings about how professionals made sense of the collaborative care model and its associated elements when initiating the intervention. Neither did the studies report much on potential conflicting interpretations of the model among the professionals. However, some studies reported on insufficient understanding of the collaborative care model among participants, especially in primary care [9, 34, 37, 39]. For instance, in the study by Eghaneyan et al. [34], some participants “described a lack of understanding about how the model worked, and more specifically, of the role of the Care Manager” (p. 508) and some were surprised by the changes required to implement the collaborative care model. In the study by Nutting et al. [30], most of the clinicians were unable to “distinguish self-management support from routine patient education about depression, despite emphasis on self-management support in the physician training” (p. 131). Further, in the study by Coupe et al. [9], “the majority of GP [general practitioner] respondents did not fully understand the CC [collaborative care] framework and could not differentiate between the management of patients with depression in CC as distinct from routine care.” In contrast, the CMs in this study had a good understanding of the collaborative care framework due to their training [9]. In the study by Wozniak et al. [31], some of the professionals (physicians as well as CMs and specialists) were not confident about practicing collaborative care due to the unfamiliarity of the model. Consequently, most of these studies emphasized the importance of educational programs which provide all participants with a thorough introduction to the principles and tools of collaborative care. In the study by Sanchez et al. [35], the use of a physician champion (along with early “identification and treatment of the most problematic patients”) was key to helping the primary care team understand the model. In the same vein, other studies [29, 40, 42] found it important to clarify roles and responsibilities between the participants from primary and secondary care.

### Cognitive participation

Not surprisingly, the question of buy-in among the professionals was presented as important in several studies. Some studies reported that the engagement of the professionals was a critical enabler for successful implementation [32, 34, 40], and a lack of engagement among the PCPs was often cited as a barrier to implementation [9, 31–34, 37]. The studies presented various explanations for skepticism and problems with engagement among PCPs:

In several studies, respondents pointed to time pressure and competing priorities in primary care [9, 30, 33, 38, 43] and problems with reimbursement [38].

Further, respondents in some studies [32, 34, 39] speculated that not all PCPs were comfortable with (or interested in) diagnosing and treating mental health illness, or that they thought they had too few relevant patients (with anxiety) to include in the collaborative care program [32].

In the study by Wozniak et al. [31], some of the non-PCP respondents speculated that PCPs may have had concerns about their clinical autonomy and status in the professional hierarchy due to increased interference from CMs and psychiatric specialists. Also, Sanchez et al. [35] mentioned that some of the participating professionals from primary care had protested about sharing the private health information of patients contained in the medical records. However, such concerns about autonomy and data privacy were not mentioned in the remainder of the included studies.

A number of studies reported that professional engagement was strengthened by the observation or communication of positive outcomes for the patients in the intervention [32, 38, 41] and that professional opinion leaders or local champions facilitated implementation [32, 35, 40]. In the study by Palinkas et al. [39], skepticism about medication among clinic staff was eased when the psychiatrist stepped in to assist. However, Curran et al. [32] found that it was difficult to increase physicians’ engagement when they were not motivated in advance. It was also difficult for practice nurses to act as local champions since they had a peripheral role in the collaboration which mostly involved the PCP and the CM [32]. Related to the issue of reimbursement mentioned in Nutting et al. [38], Whitebird et al. [40] found it important that the operating costs of collaborative care were somehow covered, so as not to be regarded as a problem by the PCPs.

### Collective action

#### Co-location and regular interaction

Co-location of the CM and the PCP in the primary care clinic was emphasized as an important enabler for implementing collaborative care in several studies [6, 9, 29, 32, 37–40] as co-location increased opportunities for regular face-to-face interaction (formal and informal) between the CM and the PCP:

it’s just so much easier. She can stop me here immediately when she has a question, and we just hand the charts back and forth. We don’t have to have separate forms, … plus, we’ve found the patients very, very accepting of it when I see them and I prescribe a drug and I say, ‘[care manager name] is going to call you and see how you’re doing.’ They know who it is and there doesn’t have to be a lot of explanation or permission or anything. (clinician cited in [38] p. 35)

Regular face-to-face interaction (e.g., at formal supervision meetings and/or more informally during lunchtime) was cited as critical for collaboration between the professionals [9, 29, 32, 38], and according to Byng et al. [33], it was important that this interaction centered on specific patient cases. Likewise, Whitebird et al. [40] found it important that referrals of patients from clinician to care manager were mostly “conducted face-to-face rather than through indirect means” (i.e., “warm hand-off”) (p. 701).

Just as co-location and regular interaction were described as enabling implementation, their absence was described as a barrier [9, 32–34], and in the study by Wozniak et al. [31], the respondents “advised having face-to-face collaboration between the CMs and family physicians (i.e. co-location) rather than the centralized model implemented” (p. 588). However, Palinkas et al. [39] and Curran et al. [32] reported that the provision of space for additional staff was a challenge for some clinics; while Wozniak et al. [31] found that discontinuity in the CM role (e.g., due to staff turnover) made it more difficult for CMs to create good working relationships with PCPs, patients and MHSs.

#### IT systems

Systems of information technology (IT) were mostly described as barriers to implementation which hindered effective communication between actors [9, 29, 34, 37, 39]. Different systems could be difficult to integrate [34], difficult to work with/for CMs [9], could lead to double registration [29], and in some interventions, the CM had limited access to the PCP’s IT system [9, 37]. Only one study mentioned IT as something which facilitated communication [36], and one study found that IT supported the monitoring of patients [29]. Generally, the studies did not describe the technical/organizational reasons for these problems in much detail.

#### The skills of the CMs

Several studies found the professional and social skills of the CMs to be an important enabler of implementation since this strengthened their organizational position in the primary care clinic [29, 32–34, 40]. Positive features of the CMs emphasized by the primary care professionals included experience [33], ability to build relationships [34], and being engaging and visible [32]. Authors also highlighted the importance of good educational programs which prepared CMs for their role [29, 33, 34, 40]. As noted in one study: “Many DCSs [CMs] had prior formal mental health training. For those who did not, additional general mental health content would have been valuable” [29]. In the case study by Sanchez et al. [35], the CM was a clinical social worker and experienced a “difficult initiation period, as she believed the primary care physicians wanted a psychiatrist or a psychiatric nurse in the role of care manager.” But by proving herself in the treatment of some of the most challenging patients, she managed to win the trust of the physicians.

#### The patient encounter

Overall, the studies reported relatively few critical barriers related to delivering the therapeutic elements of collaborative care in the interaction with patients. However, some barriers were mentioned. In the study by Eghaneyan et al. [34], primary care staff had some difficulty managing mental health problems due to the multifaceted nature of problems that patients present in primary care. In the study by Huang et al. [36], the CMs reported that some patients could be difficult to engage and some had problems which were too severe for the CMs to handle. Coupe et al. [9] described how CMs and supervisors had “some problems around delivering the trial psychological intervention (BA) in line with the protocol for those with comorbid mental health and complex social problems.” Also, in the study by Knowles et al. [6], where the collaborative care intervention explicitly aimed to integrate the treatment of mental and physical health problems, this integration was often difficult to achieve in consultations due to patient preferences for keeping such issues separated.

Regarding enablers for delivering collaborative care to patients, some studies [29, 36, 39, 42] emphasized the importance of professionals being able to engage with patients and this could be done in various ways (empathy, language, starting out with the most simple treatment strategies, making patients experience that time was available). Standardized instruments for including patients in collaborative care, for keeping track of progress, and for planning support were also seen to facilitate implementation since professionals reported having good experiences with using such instruments [29, 30, 34, 35].

#### Time and workload

The issue of time and workload in collaborative care was described and experienced in various ways by the respondents across the studies. The general message was that time is a critical factor when implementing collaborative care. According to Curran et al. [32], “[p]erhaps the most universal facilitator across clinic stakeholders was that the CALM intervention was not overly burdensome,” and in Coupe et al., the respondents viewed time as “the biggest resource necessary to implement CC, because of the time needed to maintain the prompt commencement of the intervention following referral, for the time required for the administration involved in communicating with GPs, and the time invested in supervision.” In some studies, the extra time needed for making collaborative care work (especially due to increased communication between participants) was described as a problem by PCPs [9, 30, 38, 39]. According to Nutting et al. [30], some of the PCPs “indicated that their daily schedule was a ‘zero sum game’ and that adding additional tasks to improve depression care would inevitably mean that other tasks would fall off the table” (p. 132). However, in other studies, collaborative care was not perceived to be a problem in terms of workload [32, 41, 43], and some PCPs even experienced that the model reduced their workload [32, 43]. The reasons for these differences between studies (and between PCP respondents in the same studies) were difficult to untangle. A few studies [32, 41] noted that implementation of collaborative care was facilitated in settings where PCPs were already used to working with “physician extenders” [41] such as CMs. When listing a number of organizational features that appeared to facilitate implementation (such as a shared vision, a clear change strategy, and durability of leadership), Nutting et al. [30] also emphasized “an ability within the organization to rationalize the cost of” collaborative care (p. 134). In studies addressing the sustainability of implementation, funding for collaborative care after the grant period was mentioned as a critical factor [32, 39, 41].

### Reflexive monitoring

Reflexive monitoring concerns the opportunities for evaluating the consequences of a new intervention and the possible changes in actors’ perceptions and behavior that this evaluation brings about. In collaborative care, the professionals have several ways to assess the consequences of the intervention for patients: through observations during clinical encounters, via some form of feedback from patients (e.g., where the patients tell the PCP about how things are going with the CM), at supervision meetings where the CM, PCP, and MHS can review specific patient cases, and/or through systematic monitoring of clinical outcomes at individual and aggregated levels.

The professionals’ access to information varied between studies and sometimes between local settings in the same study. In studies that reported on perceptions of patient progress, most professionals believed that patients benefitted positively from collaborative care (especially in [31, 32, 38, 39], and to a lesser degree in [9]). Curran et al. [32] reported that provider motivation had increased during the intervention as “reduced somatic complaints were observed in some patients.” Nutting et al. [38] and Sanchez et al. [35] described clinicians who had at first expressed reservations about collaborative care but then became more positive as they experienced how the CM contributed to patient treatment and support.

Several studies reported that systematic feedback on the patients’ conditions was valued by the primary care providers [29, 32, 33, 38], and that systematic monitoring enabled active follow-up thereby strengthening the implementation of collaborative care [29]. Elaborating on this issue, Byng et al. [33] showed that collaborative care suffered in clinics that did not manage to set up systems for monitoring patients’ progress [33]. Furthermore, Palinkas et al. [39] found that even though clinicians were generally of the opinion that patients benefitted from the intervention, they were nevertheless “unsure of the value of continuing the services in the absence of immediate access to objective data” [39]. Likewise, in the study by Coupe et al. [9], “some GPs suggested that the results of the trial rather than their views would determine their opinions on the future possibility of working in a new way.” When monitoring patient outcomes in collaborative care for anxiety and depression, an instrument (such as PHQ-9) for assessing the severity of mental illness is employed. Studies reporting on this subject found that professionals generally valued using such instruments to monitor patient progress [30, 34–36] (Table 2).

**Table 2:** Overview of findings related to the four dimensions of Normalization Process Theory (NPT)

NPT-dimensions	Enablers	Barriers
Coherence	Training [9] Physician champion [35] Clarification of roles and responsibilities among professionals [40, 42, 29]	Lack of educational programs [31]
Cognitive participation	Professionals made aware of positive patient outcomes [41, 32, 38] Local opinion leaders [32, 35, 40] Covering PCPs operating costs related to collaborative care [40] Psychiatric supervision can ease scepticism among staff about medication [39]	Lack of engagement among the PCPs [33, 9, 32, 34, 37, 31] Time pressures [33, 9, 43, 38, 30] Problems with reimbursement [38] PCPs being uncomfortable with diagnosing and treating mental health illness [32, 34, 39] PCP concerns about sharing patients’ private health information [35]
Collective action	Co-location of CM and PCP [9, 32, 37, 29, 39, 38, 40, 6] Regular face-to-face interaction between professionals [9, 32, 38, 29] Interaction between professionals being centered on patient cases [33] Face-to-face patient referral between professionals [40] Professionals able to engage with patients [36, 42, 39, 29] CMs’ social and professional skills, e.g. being visible, able to build relationships [33, 32, 34, 29, 40] Good educational programs for CMs [33, 34, 29, 40] Model not being burdensome or create a problems with workload [32, 41, 43] Instruments for including patients and keeping track of progress [34, 29, 35, 30]	Absence of co-location of CM and PCP [33, 9, 32, 34] Lack of space for additional staff [39, 32] Difficulties engaging patients due to patients’ problems being too severe or complex [9, 36] and/or due to patients’ preferences [6] Primary care staff having difficulties managing mental health problems [34] Making the model work experienced as consuming [39, 9, 30, 38] IT-systems hindered effective communication (e.g. double registration, limited access, lack of integration) [9, 37, 29, 39, 34].
Reflexive monitoring	Professionals experience that patients benefit from collaborate care [32, 35, 38] Primary care providers value systematic patient feedback [33, 32, 29, 38] and instruments for monitoring patient progress [34, 36, 35, 30] Systematic monitoring enable active follow up which strengthen implementation [29]	Lack of systems for monitoring patient progress [33] Absence of immediate access to objective data on patient progress [39]

## Discussion

To our knowledge, this is the first systematic review of qualitative studies about the barriers and enablers to implementing collaborative care for mental illness. The review included 17 studies almost all of which were conducted in the USA (*n* = 11) and the UK (*n* = 5). While most studies relied on some form of interview as their data collection method, there was much heterogeneity across studies concerning study samples, research focus, analytical approaches, and depth of analysis. The review identified several barriers and enablers within the four major analytical dimensions of NPT with the studies paying the most attention to issues related to cognitive participation and collective action. The studies generally agreed that securing buy-in among PCPs was a critical but sometimes difficult task when introducing collaborative care interventions. Various explanations for the lack of engagement (cognitive participation) among PCPs were offered in the studies such as a lack of confidence or interest in treating mental illness along with time pressure and competing priorities in primary care. Some enablers for PCP engagement such as reimbursement (covering the expenses of implementation work), physician champions, and feedback on the effectiveness of collaborative care were also presented.

Several studies reported that professionals were quite positive after having experienced collaborative care. This indicates that initial skepticism among PCPs toward the concept of collaborative care is not necessarily fundamental or persistent but may be overcome. One factor that seemed critical for winning acceptance for collaborative care was that the CM proved to be socially and professionally competent in the eyes of the primary care providers. Another significant theme across studies concerned the location of the care manager in relation to primary care. Several studies found that co-location of the PCP and the CM was an important facilitator for the daily enactment of collaborative care (collective action)— since co-location supported regular face-to-face interaction between PCP and CM. Face-to-face interaction may facilitate both common understandings of collaboration, case discussions, and attempts to optimize collaborative efforts. These results suggest that the NPT concept of relational integration (a sub-construct of collective action) [11] is particularly relevant for understanding and improving the implementation of collaborative care since relational integration points to the importance of assuring that professionals develop and maintain trust in the intervention and in each others’ contributions.

### Strengths and limitations

#### Strengths and limitations relating to the methodology of the review

This review has several methodological strengths: first, it was conducted by a multidisciplinary research team (GO is a language psychologist, ASD is an experienced general practitioner, and MBK is a political scientist) all well-rehearsed in qualitative methods. Second, study selection, quality assessment, and analysis were performed by at least two of the authors in order to strengthen reliability. Third, we executed a relatively comprehensive search in five major databases with the assistance of an experienced research librarian. Fourth, we used the ENTREQ standards for reporting qualitative reviews, the COREQ standards for quality assessment, and the theoretical implementation framework offered by NPT for data analysis—all of which have previously been applied in reviews of qualitative studies.

However, some limitations still apply: first, since we wanted only to include peer-reviewed studies, we did not search the gray literature, and for resource reasons, we did not snowball search the selected studies for further material. Hence, some relevant studies may have been missed. Second, the use of quality standards does not necessarily guarantee the quality of intellectual work where interpretation is essential. More specifically, while COREQ does capture several relevant aspects of quality, in our opinion, it also has some limitations as an assessment tool due to its strong focus on the formal aspects of research papers and its relatively weak focus on analytical content. Also, assessing each criterion in a yes/no format (which is how COREQ is often applied in systematic reviews, including this one) can be difficult for some of the quality criteria in COREQ. For example, while it is relatively easy to assess whether a paper contains a description of the sampling method or not, the assessment of criteria such as clarity of minor themes or consistency of data and results is more difficult since a paper may contain detailed and consistent descriptions of some themes but be more superficial or inconsistent concerning others. In future reviews, a slightly more differentiated scale could be considered when assessing individual quality criteria.

Concerning the use of NPT, it was relatively easy to fit the findings within the overall theoretical framework, but it was sometimes quite difficult to decide exactly how a particular finding should be categorized since some findings seemed to cut across theoretical constructs. Such problems with overlap in NPT analysis have previously been noted [46].

#### Strengths and limitations relating to the included studies

The studies in this review provided several interesting findings about the challenges and possibilities of implementing collaborative care. Several studies included a large and varied number of respondents and the average COREQ score of 15.5 in the quality assessment was about equal to that found in other reviews of qualitative studies [26–28].

However, some important limitations in the set of included studies should be taken into consideration.

First, almost all of the studies (16/17) were conducted in the USA (*n* = 11) or the UK (*n* = 5). The significant differences in health systems between these countries render direct comparisons difficult. The predominance of studies from these two countries also limits the generalisability of the findings to other national health-care settings. Second, most studies were linked to collaborative care interventions focused on depression. Issues related to implementing collaborative care for anxiety are therefore not explored to the same degree. Third, there were some problems with study quality: in the COREQ, assessment only seven studies (out of 17) fulfilled more than half of the 32 COREQ criteria, although the lowest scores were found in domain 1 which we do not consider to be as important as the two other domains. Also, a few studies included findings which were not particularly well validated (e.g., barriers among PCPs) and several studies did not include respondents from all of the key professional groups involved in the collaborative care (PCPs *and* CMs *and* MHSs). Since the identification and articulation of barriers and enablers is affected by the perspectives and resources of all the participating actors [47] and since several types of actors are, by definition, involved in collaborative care, it seems important to include respondents from all of the key professional groups when exploring implementation. Some studies did not describe the identified barriers and facilitators in much detail. The lack of thick description has previously been noted in qualitative studies published in health-care journals where space is often limited [48], and although several important themes could be identified, the results could not always be easily compared or condensed, due to the limited and varied descriptions of interventions and contexts and due to variation in the types of barriers and enablers reported. The variation in the types of barriers and enablers identified could suggest that some studies were not sufficiently comprehensive in exploring these issues or it could be ascribed to the strong influence of the specific intervention design and the local context on the implementation process (and hence on the barriers and enablers experienced in each intervention). Finally, almost all studies relied on interview data, and none used participant observations. Although these can be costly and impractical, they can strengthen the validity of findings by going beyond the participants’ interview statements.

## Conclusion

The results from this review point to several important implications for policy and management when implementing collaborative care models. First, special notice should be given to designing an effective educational program to prepare CMs for their central professional and organizational role in collaborative care in relation to PCPs, MHSs, and patients. The implementation strategy should also include a plan for providing PCPs with a thorough understanding of the collaborative care model and what it requires of them. Arrangements should be made with primary care providers regarding reimbursement for extra work and expenses associated with collaborative care [49]. Next, robust systems for communication and monitoring should be in place before implementation begins. Furthermore, the results suggest that collaborative care programs should be designed to include regular face-to-face interaction especially in relation to hand-offs and case discussions of patients between the CM and the responsible PCP. In order to facilitate informal and formal interaction, the CM should preferably be located in the relevant primary care clinics. In settings where co-location is not an option, other possibilities for face-to-face interaction should be considered. Since the implementation of quality improvement concepts always unfolds in a particular social, political, and financial context, it is up to the initiating actors (e.g., policymakers, managers, quality improvement agencies, and researchers) to tailor the specific model of collaborative care and the strategy for its implementation to the target group and the available resources. With regard to future research, this review has identified a number of limitations in the current knowledge base pointing to a need for well-sampled, in-depth, qualitative studies of the implementation of collaborative care in health settings outside the USA and the UK.

## Additional files

Additional file 1: PubMed search string and list of ProQuest databases used. (DOCX 14 kb)
Additional file 2: COREQ checklist. (DOCX 18.4 KB)

## Abbreviations

CM: care manager
COREQ: consolidated criteria for reporting qualitative research
ENTREQ: enhancing transparency in reporting the synthesis of qualitative research
IT: information technology
MHS: mental health specialist
NPT: normalization process theory
PCP: primary care physician
