# Supplementary material for: Enablers and barriers to implementing collaborative care for anxiety and depression: a systematic qualitative review
Source: Implement Sci. 2016 Dec 28;11:165. doi: 10.1186/s13012-016-0519-y (PMC5192575; doi:10.1186/s13012-016-0519-y)
Supplement: Additional file 1: — PubMed search string and list of ProQuest databases used. (DOCX 14 kb) [file 13012_2016_519_MOESM1_ESM.docx]

**Additional file 1: PubMed search string and list of ProQuest databases used**

((((((("collaborative care" OR "shared care" OR "Delivery of Health Care, Integrated"[Mesh:noexp] OR "integrated care" OR "integrated health care" OR "integrated patient care" OR "case management"[Mesh] OR "case management" OR "case manager" OR "case managers"))) OR ("care management" OR "care manager" OR "care managers"))) AND (("Anxiety Disorders"[Mesh] OR "Depression"[Mesh] OR "Depressive Disorder"[Mesh] OR Anxiety OR Depression OR "Mental Disorders"[Mesh] OR "mental disorder" OR "mental disorders" OR "mental illness" OR "mental disease"))) AND ((implement* OR "Organization and Administration"[Mesh] OR "Leadership"[Mesh] OR "Patient Care Management"[Mesh:noexp] OR "Personnel Management"[Mesh]))) AND (("Qualitative Research"[Mesh] OR "qualitative research" OR "qualitative study" OR "qualitative studies" OR "qualitative evaluation" OR "qualitative interview" OR "qualitative interviews" OR "Focus Groups"[Mesh] OR "focus groups" OR "focus group" OR "process evaluation" OR "ethnographic study" OR "ethnographic studies"))

[ProQuest Family Health](http://search.proquest.com.ep.fjernadgang.kb.dk/familyhealth/fromDatabasesLayer?accountid=13607)

[ProQuest Health & Medical Complete](http://search.proquest.com.ep.fjernadgang.kb.dk/healthcomplete/fromDatabasesLayer?accountid=13607)

[ProQuest Health Management](http://search.proquest.com.ep.fjernadgang.kb.dk/healthmanagement/fromDatabasesLayer?accountid=13607)

[ProQuest Nursing & Allied Health Source](http://search.proquest.com.ep.fjernadgang.kb.dk/nursing/fromDatabasesLayer?accountid=13607)

[ProQuest Psychology Journals](http://search.proquest.com.ep.fjernadgang.kb.dk/psychology/fromDatabasesLayer?accountid=13607)

[ProQuest Social Science Journals](http://search.proquest.com.ep.fjernadgang.kb.dk/socscijournals/fromDatabasesLayer?accountid=13607)

[ProQuest Sociology](http://search.proquest.com.ep.fjernadgang.kb.dk/sociology/fromDatabasesLayer?accountid=13607) (1985 - current)

[ProQuest Dissertations & Theses A&I‎](http://search.proquest.com.ep.fjernadgang.kb.dk/pqdt/dissertations/fromDatabasesLayer?accountid=13607)

[ProQuest Dissertations & Theses Global‎](http://search.proquest.com.ep.fjernadgang.kb.dk/pqdtglobal/dissertations/fromDatabasesLayer?accountid=13607)

[ProQuest Social Science Journals‎](http://search.proquest.com.ep.fjernadgang.kb.dk/socscijournals/socialsciences/fromDatabasesLayer?accountid=13607)
