# Supplementary material for: Enablers and barriers to implementing collaborative care for anxiety and depression: a systematic qualitative review
Source: Implement Sci. 2016 Dec 28;11:165. doi: 10.1186/s13012-016-0519-y (PMC5192575; doi:10.1186/s13012-016-0519-y)
Supplement: Additional file 2: — COREQ checklist. (DOCX 18.4 KB) [file 13012_2016_519_MOESM2_ESM.docx]

| **Table 2: COREQ Checklist** | | | |
| --- | --- | --- | --- |
|  | Number of studies who reported the criterion | | References to the studies |
| **Domain 1: Research team and reflexivity** | | | |
| 1. Inter viewer/facilitator | 11 | 65% | [8, 21, 23, 24, 25, 26, 27, 28, 29, 31 34,] |
| 2. Credentials | 6 | 35% | [20, 22, 26, 27, 31,35] |
| 3. Occupation | 2 | 12% | [34, 31] |
| 4. Gender | 0 | 0% |  |
| 5. Experience and training | 2 | 12% | [23,27] |
| *Relationship with participants* |  |  |  |
| 6. Relationship established | 1 | 6% | [25] |
| 7. Participant knowledge of the interviewer | 2 | 12% | [23,25] |
| 8. Interviewer characteristics | 3 | 18% | [8, 24, 30] |
| **Domain 2: study design** | | | |
| *Theoretical framework* | | | |
| 9. Methodological orientation and Theory | 14 | 82% | [8, 22, 23, 24, 25, 26, 27, 28, 30, 31, 32, 33, 34, 35] |
| *Participant selection* | | | |
| 10. Sampling | 13 | 76% | [8, 20, 21, 22, 23, 24, 25, 26, 27, 29, 30, 31, 34, 35] |
| 11. Method of approach | 15 | 88% | [8, 20, 21, 23, 24, 25, 26, 27, 28, 29, 30, 31, 33, 34, 35] |
| 12. Sample size | 16 | 94% | [8, 20, 21, 22, 23, 24, 25, 26, 27, 28, 29, 30, 32, 33, 34, 35] |
| 13. Non-participation | 8 | 47% | [20, 21, 22, 23, 26, 28, 34, 35] |
| *Setting* | | | |
| 14. Setting of data collection | 9 | 53% | [8, 21, 23, 25, 26, 27, 29, 31, 35] |
| 15. Presence of non-participants | 1 | 6% | [25] |
| 16. Description of sample | 13 | 76% | [8, 20, 21, 23, 24, 26, 27, 28, 29, 30, 32, 34, 35] |
| *Data collection* | | | |
| 17. Interview guide | 13 | 76% | [8, 20, 21, 23, 24, 25, 26, 27, 30, 31, 32, 34, 35] |
| 18. Repeat interviews | 6 | 35% | [20, 22, 28, 29 34, 35] |
| 19. Audio/visual recording | 14 | 82% | [8, 20, 21, 22, 23, 24, 25, 26, 28, 29, 32, 33, 34, 35] |
| 20. Field notes | 2 | 12% | [20, 26] |
| 21. Duration | 6 | 35% | [8, 20, 25, 26, 30, 34] |
| 22. Data saturation | 4 | 24% | [23, 25, 32, 34] |
| 23. Transcripts returned | 0 | 0% |  |
| **Domain 3: analysis and ﬁndings** | | | |
| *Data analysis* | | | |
| 24. Number of data coders | 13 | 76% | [8, 21, 22, 23, 24, 25, 26, 27, 28, 31, 32, 34, 35] |
| 25. Description of the coding tree | 9 | 53% | [8, 22, 23, 25, 29, 30, 31, 34, 35] |
| 26. Derivation of themes | 14 | 82% | [8, 20, 21, 22, 23, 24, 25, 26, 27, 28, 30, 32, 34, 35] |
| 27. Software | 5 | 29% | [21, 23, 34, 27, 29] |
| 28. Participant checking | 1 | 6% | [20] |
| *Reporting* | | | |
| 29. Quotations presented | 13 | 76% | [8, 22, 23, 24, 25, 26, 27, 28, 29, 30, 32, 34, 35] |
| 30. Data and ﬁndings consistent | 16 | 94% | [8, 20, 21, 22, 23, 24, 25, 26, 27, 28, 29, 30, 31, 32, 34, 35] |
| 31. Clarity of major themes | 17 | 100% | [8, 20, 21, 22, 23, 24, 25, 26, 27, 28, 29, 30, 31, 32, 33, 34, 35] |
| 32. Clarity of minor themes | 14 | 82% | [8, 21, 23, 24, 25, 26, 27, 28, 29, 30, 32, 33, 34, 35] |
